# (4-Amino-3-methyl­benzene­sulfonato)tri-μ-aqua-penta­aqua­disodium 4-amino-3-methyl­benzene­sulfonate

**DOI:** 10.1107/S1600536809055512

**Published:** 2010-01-09

**Authors:** Xi-Shi Tai, Fu-Gong Zhang

**Affiliations:** a College of Chemistry and Chemical Engineering, Weifang University, Weifang 261061, People’s Republic of China; b Department of Physics, Weifang University, Weifang 261061, People’s Republic of China

## Abstract

In the title compound, [Na_2_(C_7_H_8_NO_3_S)(H_2_O)_8_](C_7_H_8_NO_3_S), one Na^+^ ion is bonded to six water mol­ecules in a distorted octa­hedral arrangement while the other is bonded to five water mol­ecules and one O atom of a 4-amino-3-methyl­benzene­sulfonate anion, also yielding a distorted NaO_6_ octa­hedron. Three of the water molecules bridge the metal ions and an intra­molecular O—H⋯O hydrogen bond helps to establish the conformation. In the crystal, the component species inter­act by way of O—H⋯O, O—H⋯N and N—H⋯O hydrogen bonds.

## Related literature

For background to coordination networks, see: Tai *et al.* (2007 ▶); Wang *et al.* (2008 ▶).
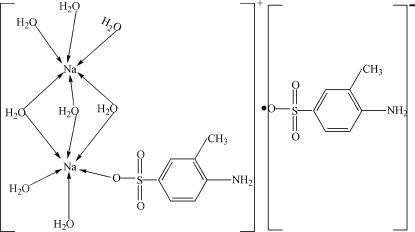


## Experimental

### 

#### Crystal data

[Na_2_(C_7_H_8_NO_3_S)(H_2_O)_8_](C_7_H_8_NO_3_S)
*M* *_r_* = 562.52
Monoclinic, 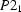

*a* = 6.2346 (17) Å
*b* = 27.793 (8) Å
*c* = 7.285 (2) Å
β = 91.296 (5)°
*V* = 1261.9 (6) Å^3^
*Z* = 2
Mo *K*α radiation
μ = 0.31 mm^−1^
*T* = 298 K
0.24 × 0.20 × 0.18 mm

#### Data collection

Bruker SMART CCD diffractometer
Absorption correction: multi-scan (*SADABS*; Bruker, 2000 ▶) *T*_min_ = 0.929, *T*_max_ = 0.946
6522 measured reflections
4067 independent reflections
3823 reflections with *I* > 2σ(*I*)
*R*_int_ = 0.034

#### Refinement

*R*[*F* ^2^ > 2σ(*F* ^2^)] = 0.038
*wR*(*F* ^2^) = 0.097
*S* = 1.05
4067 reflections
308 parameters
H-atom parameters constrained
Δρ_max_ = 0.34 e Å^−3^
Δρ_min_ = −0.33 e Å^−3^
Absolute structure: Flack (1983 ▶), 1779 Friedel pairs
Flack parameter: 0.03 (7)

### 

Data collection: *SMART* (Bruker, 2000 ▶); cell refinement: *SAINT* (Bruker, 2000 ▶); data reduction: *SAINT*; program(s) used to solve structure: *SHELXS97* (Sheldrick, 2008 ▶); program(s) used to refine structure: *SHELXL97* (Sheldrick, 2008 ▶); molecular graphics: *SHELXTL* (Sheldrick, 2008 ▶); software used to prepare material for publication: *SHELXTL*.

## Supplementary Material

Crystal structure: contains datablocks global, I. DOI: 10.1107/S1600536809055512/hb5297sup1.cif

Structure factors: contains datablocks I. DOI: 10.1107/S1600536809055512/hb5297Isup2.hkl

Additional supplementary materials:  crystallographic information; 3D view; checkCIF report

## Figures and Tables

**Table 1:** Selected bond lengths (Å)

Na1—O9	2.382 (3)
Na1—O8	2.390 (3)
Na1—O1	2.399 (3)
Na1—O7	2.453 (3)
Na1—O10	2.466 (3)
Na1—O11	2.670 (3)
Na2—O10	2.341 (3)
Na2—O13	2.348 (3)
Na2—O14	2.380 (3)
Na2—O9	2.441 (3)
Na2—O12	2.490 (3)
Na2—O11	2.516 (3)

**Table 2:** Hydrogen-bond geometry (Å, °)

*D*—H⋯*A*	*D*—H	H⋯*A*	*D*⋯*A*	*D*—H⋯*A*
N1—H1*A*⋯O4^i^	0.86	2.30	3.046 (4)	145
N2—H2*A*⋯O1^ii^	0.86	2.30	3.066 (4)	149
O7—H15⋯N2^iii^	0.85	2.13	2.929 (4)	157
O7—H16⋯O2^iv^	0.85	1.98	2.809 (3)	166
O8—H17⋯O12^v^	0.85	2.29	2.800 (4)	119
O8—H18⋯O6	0.85	2.14	2.989 (4)	171
O9—H19⋯O3^vi^	0.85	2.00	2.814 (4)	160
O9—H20⋯O8^vi^	0.85	2.06	2.911 (4)	175
O10—H21⋯O2	0.85	1.99	2.831 (3)	172
O10—H22⋯O14^vii^	0.85	2.01	2.812 (4)	157
O11—H23⋯O4	0.85	2.05	2.895 (4)	175
O11—H24⋯O5^vi^	0.85	2.03	2.872 (4)	169
O12—H25⋯O7^viii^	0.85	1.95	2.794 (3)	175
O12—H26⋯O2^vi^	0.85	2.09	2.889 (4)	157
O13—H27⋯O6^viii^	0.85	2.12	2.969 (4)	175
O13—H28⋯O5^ix^	0.85	2.32	2.883 (4)	124
O14—H29⋯O6^ix^	0.85	2.04	2.837 (3)	156
O14—H30⋯O4^vi^	0.85	1.95	2.790 (3)	171

Symmetry codes: (i) 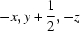; (ii) 
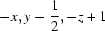
; (iii) 
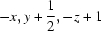
; (iv) 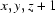; (v) 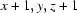; (vi) 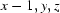; (vii) 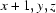; (viii) 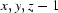; (ix) 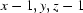.

**Fig. 1.**
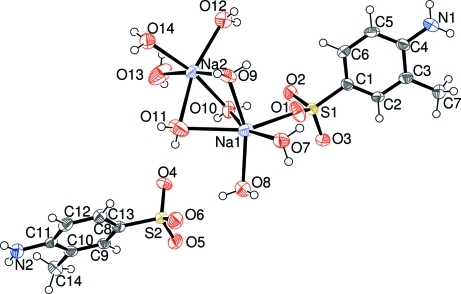
The molecular structure of (I) showing 30% displacement ellipsoids.

## supplementary crystallographic information

### Comment

During the past two decades, the design and synthesis of organic-inorganic hybrid materials have attracted intense attentions owing to their potential practical applications [Wang, *et al.*, 2008; Tai, *et al.*, 2007]. Benzenesulfonic acids can potentially be monodentate, bidentate ligands, they may also be intermolecularly bridging or intramolecularly chelating ligands. As part of our ongoing study in this field, In this paper, we report on the synthesis and crystal structure of the title compound, (I), (Scheme I).

In the crystal of (I), Na1 atom is six coordinate to five water molecules and one ligand, and Na2 atom is six coordinate to water molecules. The bond distances of Na—O are in the range of 2.341 (3)–2.670 (3). Otherwise, the geometrical parameters for (I) are normal. In the crystal packing, the molecules form a thrre-dimensional network by hydrogen bonds.

### Experimental

A solution of 1.0 mmol NaOH in 1 ml water was added to a solution of 1.0 mmol 4-amino-3-methyl-benzenesulfonic acid in 5 ml ethanol at room temperature. The mixture was refluxed for 4 h with stirring, then the resulting precipitate was filtered, washed, and dried *in vacuo* over P_4_O_10_ for 48 h. Colourless blocks of (I) were obtained by slowly evaporating from methanol at room temperature.

### Crystal data

**Table:**

[Na_2_(C_7_H_8_NO_3_S)(H_2_O)_8_](C_7_H_8_NO_3_S)	*F*(000) = 592
*M**_r_* = 562.52	*D*_x_ = 1.480 Mg m^−^^3^
Monoclinic, *P*2_1_	Mo *K*α radiation, λ = 0.71073 Å
Hall symbol: P 2yb	Cell parameters from 3297 reflections
*a* = 6.2346 (17) Å	θ = 2.9–26.0°
*b* = 27.793 (8) Å	µ = 0.31 mm^−^^1^
*c* = 7.285 (2) Å	*T* = 298 K
β = 91.296 (5)°	Block, colourless
*V* = 1261.9 (6) Å^3^	0.24 × 0.20 × 0.18 mm
*Z* = 2	

### Data collection

**Table:**

Bruker SMART CCD diffractometer	4067 independent reflections
Radiation source: fine-focus sealed tube	3823 reflections with *I* > 2σ(*I*)
graphite	*R*_int_ = 0.034
ω scans	θ_max_ = 25.0°, θ_min_ = 1.5°
Absorption correction: multi-scan (*SADABS*; Bruker, 2000)	*h* = −6→7
*T*_min_ = 0.929, *T*_max_ = 0.946	*k* = −32→33
6522 measured reflections	*l* = −8→8

### Refinement

**Table:**

Refinement on *F*^2^	Secondary atom site location: difference Fourier map
Least-squares matrix: full	Hydrogen site location: inferred from neighbouring sites
*R*[*F*^2^ > 2σ(*F*^2^)] = 0.038	H-atom parameters constrained
*wR*(*F*^2^) = 0.097	*w* = 1/[σ^2^(*F*_o_^2^) + (0.0555*P*)^2^ + 0.0081*P*] where *P* = (*F*_o_^2^ + 2*F*_c_^2^)/3
*S* = 1.05	(Δ/σ)_max_ = 0.001
4067 reflections	Δρ_max_ = 0.34 e Å^−^^3^
308 parameters	Δρ_min_ = −0.32 e Å^−^^3^
0 restraints	Absolute structure: Flack (1983), 1779 Friedel pairs
Primary atom site location: structure-invariant direct methods	Flack parameter: 0.03 (7)

### Special details

**Table:**

Geometry. All e.s.d.'s (except the e.s.d. in the dihedral angle between two l.s. planes) are estimated using the full covariance matrix. The cell e.s.d.'s are taken into account individually in the estimation of e.s.d.'s in distances, angles and torsion angles; correlations between e.s.d.'s in cell parameters are only used when they are defined by crystal symmetry. An approximate (isotropic) treatment of cell e.s.d.'s is used for estimating e.s.d.'s involving l.s. planes.
Refinement. Refinement of *F*^2^ against ALL reflections. The weighted *R*-factor *wR* and goodness of fit *S* are based on *F*^2^, conventional *R*-factors *R* are based on *F*, with *F* set to zero for negative *F*^2^. The threshold expression of *F*^2^ > σ(*F*^2^) is used only for calculating *R*-factors(gt) *etc*. and is not relevant to the choice of reflections for refinement. *R*-factors based on *F*^2^ are statistically about twice as large as those based on *F*, and *R*- factors based on ALL data will be even larger.

### Fractional atomic coordinates and isotropic or equivalent isotropic displacement parameters (Å^2^)

**Table:**

	*x*	*y*	*z*	*U*_iso_*/*U*_eq_	
Na1	0.0286 (2)	0.64325 (5)	0.33149 (17)	0.0375 (3)	
Na2	−0.2402 (2)	0.58785 (5)	−0.01371 (18)	0.0375 (3)	
S1	0.27757 (12)	0.73351 (2)	0.04923 (10)	0.02893 (18)	
S2	0.40036 (12)	0.48468 (3)	0.47552 (10)	0.02884 (18)	
O1	0.1361 (4)	0.71695 (8)	0.1890 (3)	0.0498 (7)	
O2	0.2497 (4)	0.70581 (8)	−0.1205 (3)	0.0405 (6)	
O3	0.5005 (4)	0.73391 (9)	0.1084 (4)	0.0538 (7)	
O4	0.3033 (4)	0.49845 (8)	0.2983 (3)	0.0407 (6)	
O5	0.6316 (3)	0.48784 (9)	0.4768 (3)	0.0446 (6)	
O6	0.3058 (4)	0.51229 (9)	0.6233 (3)	0.0433 (6)	
O7	−0.0541 (4)	0.69570 (8)	0.5914 (3)	0.0420 (6)	
H15	−0.0754	0.7245	0.5563	0.063*	
H16	0.0534	0.6972	0.6651	0.063*	
O8	0.3432 (4)	0.61320 (9)	0.4858 (4)	0.0496 (6)	
H17	0.3231	0.6299	0.5817	0.074*	
H18	0.3269	0.5837	0.5135	0.074*	
O9	−0.3362 (4)	0.64448 (9)	0.2270 (3)	0.0407 (5)	
H19	−0.3594	0.6741	0.2060	0.061*	
H20	−0.4356	0.6355	0.2972	0.061*	
O10	0.1122 (4)	0.61615 (8)	0.0188 (3)	0.0418 (6)	
H21	0.1642	0.6418	−0.0257	0.063*	
H22	0.1900	0.5937	−0.0226	0.063*	
O11	−0.0947 (4)	0.55211 (10)	0.2810 (4)	0.0582 (7)	
H23	0.0249	0.5374	0.2812	0.087*	
H24	−0.1852	0.5327	0.3253	0.087*	
O12	−0.3884 (4)	0.64884 (9)	−0.2325 (3)	0.0454 (6)	
H25	−0.2919	0.6634	−0.2915	0.068*	
H26	−0.4664	0.6701	−0.1829	0.068*	
O13	−0.1241 (5)	0.52949 (13)	−0.2223 (5)	0.0774 (10)	
H27	0.0002	0.5263	−0.2663	0.116*	
H28	−0.2006	0.5392	−0.3130	0.116*	
O14	−0.5711 (4)	0.54510 (9)	−0.0209 (3)	0.0397 (5)	
H30	−0.6070	0.5281	0.0704	0.060*	
H29	−0.5958	0.5274	−0.1142	0.060*	
N1	0.0297 (6)	0.93420 (11)	−0.1088 (5)	0.0517 (8)	
H1A	−0.0957	0.9406	−0.1539	0.062*	
H1B	0.1188	0.9571	−0.0854	0.062*	
N2	0.1911 (5)	0.28066 (10)	0.6182 (4)	0.0353 (6)	
H2A	0.0701	0.2731	0.6648	0.042*	
H2B	0.2832	0.2586	0.5950	0.042*	
C1	0.2063 (5)	0.79329 (10)	−0.0007 (4)	0.0281 (7)	
C2	0.3493 (5)	0.83016 (11)	0.0335 (4)	0.0270 (6)	
H2	0.4857	0.8230	0.0802	0.032*	
C3	0.2944 (5)	0.87740 (11)	−0.0001 (4)	0.0294 (7)	
C4	0.0878 (5)	0.88746 (11)	−0.0741 (4)	0.0312 (7)	
C5	−0.0519 (5)	0.84973 (12)	−0.1103 (4)	0.0336 (7)	
H5	−0.1866	0.8564	−0.1615	0.040*	
C6	0.0025 (5)	0.80289 (11)	−0.0727 (4)	0.0309 (7)	
H6	−0.0948	0.7781	−0.0950	0.037*	
C7	0.4487 (6)	0.91779 (12)	0.0394 (5)	0.0387 (8)	
H7A	0.5831	0.9048	0.0832	0.058*	
H7B	0.3908	0.9386	0.1312	0.058*	
H7C	0.4709	0.9358	−0.0710	0.058*	
C8	0.3372 (5)	0.42400 (10)	0.5119 (4)	0.0283 (7)	
C9	0.4831 (5)	0.38849 (11)	0.4721 (4)	0.0254 (6)	
H9	0.6147	0.3969	0.4240	0.031*	
C10	0.4370 (5)	0.34040 (11)	0.5026 (4)	0.0277 (6)	
C11	0.2378 (5)	0.32860 (11)	0.5801 (4)	0.0277 (6)	
C12	0.0921 (5)	0.36457 (12)	0.6162 (4)	0.0314 (7)	
H12	−0.0397	0.3565	0.6648	0.038*	
C13	0.1375 (5)	0.41236 (12)	0.5819 (4)	0.0314 (6)	
H13	0.0371	0.4362	0.6049	0.038*	
C14	0.5927 (6)	0.30165 (12)	0.4548 (5)	0.0375 (7)	
H14A	0.7087	0.3153	0.3878	0.056*	
H14B	0.5211	0.2778	0.3805	0.056*	
H14C	0.6481	0.2870	0.5653	0.056*	

### Atomic displacement parameters (Å^2^)

**Table:**

	*U*^11^	*U*^22^	*U*^33^	*U*^12^	*U*^13^	*U*^23^
Na1	0.0358 (7)	0.0437 (7)	0.0328 (7)	0.0021 (6)	−0.0066 (5)	−0.0007 (6)
Na2	0.0350 (7)	0.0406 (7)	0.0366 (7)	−0.0023 (6)	−0.0043 (5)	−0.0048 (6)
S1	0.0361 (4)	0.0239 (3)	0.0265 (4)	0.0027 (3)	−0.0054 (3)	−0.0012 (3)
S2	0.0297 (4)	0.0278 (4)	0.0287 (4)	−0.0027 (3)	−0.0055 (3)	0.0001 (3)
O1	0.0738 (18)	0.0348 (13)	0.0415 (14)	0.0064 (12)	0.0198 (13)	0.0056 (11)
O2	0.0600 (15)	0.0307 (12)	0.0305 (12)	0.0035 (11)	−0.0063 (11)	−0.0075 (10)
O3	0.0416 (14)	0.0349 (12)	0.0834 (19)	0.0065 (11)	−0.0289 (13)	0.0003 (14)
O4	0.0497 (14)	0.0397 (13)	0.0322 (12)	0.0006 (10)	−0.0117 (10)	0.0122 (10)
O5	0.0300 (12)	0.0409 (13)	0.0627 (16)	−0.0061 (11)	−0.0063 (10)	0.0021 (13)
O6	0.0531 (14)	0.0388 (13)	0.0379 (13)	−0.0005 (11)	−0.0008 (11)	−0.0086 (11)
O7	0.0507 (14)	0.0400 (13)	0.0349 (13)	0.0013 (11)	−0.0080 (11)	0.0030 (11)
O8	0.0577 (16)	0.0386 (13)	0.0517 (16)	−0.0001 (12)	−0.0126 (12)	0.0028 (12)
O9	0.0391 (12)	0.0406 (13)	0.0421 (13)	0.0112 (10)	−0.0043 (10)	0.0037 (11)
O10	0.0422 (13)	0.0299 (11)	0.0534 (16)	−0.0051 (10)	0.0052 (11)	−0.0034 (11)
O11	0.0482 (15)	0.0604 (17)	0.0660 (18)	0.0090 (13)	0.0051 (13)	0.0274 (15)
O12	0.0454 (14)	0.0483 (14)	0.0427 (13)	0.0031 (12)	0.0030 (11)	−0.0014 (11)
O13	0.0479 (16)	0.104 (3)	0.080 (2)	0.0001 (17)	0.0021 (15)	−0.050 (2)
O14	0.0467 (14)	0.0394 (12)	0.0329 (13)	−0.0018 (11)	0.0004 (10)	0.0006 (10)
N1	0.055 (2)	0.0352 (16)	0.064 (2)	0.0056 (14)	−0.0221 (16)	0.0098 (15)
N2	0.0368 (15)	0.0323 (14)	0.0366 (15)	−0.0062 (12)	−0.0018 (12)	0.0027 (12)
C1	0.0340 (16)	0.0256 (16)	0.0246 (14)	0.0029 (12)	−0.0028 (13)	0.0007 (12)
C2	0.0257 (15)	0.0290 (15)	0.0259 (15)	0.0005 (12)	−0.0064 (12)	0.0037 (12)
C3	0.0305 (16)	0.0353 (17)	0.0222 (15)	−0.0003 (13)	−0.0036 (12)	0.0028 (12)
C4	0.0368 (17)	0.0301 (15)	0.0264 (16)	0.0069 (13)	−0.0052 (13)	0.0030 (13)
C5	0.0268 (16)	0.0410 (18)	0.0325 (17)	0.0060 (13)	−0.0105 (13)	0.0001 (14)
C6	0.0301 (16)	0.0319 (15)	0.0302 (16)	−0.0031 (13)	−0.0070 (13)	−0.0043 (13)
C7	0.0456 (19)	0.0301 (17)	0.0401 (19)	−0.0033 (15)	−0.0080 (15)	0.0004 (15)
C8	0.0314 (16)	0.0313 (16)	0.0220 (14)	−0.0033 (12)	−0.0052 (12)	0.0027 (12)
C9	0.0252 (14)	0.0327 (15)	0.0183 (14)	−0.0018 (12)	−0.0020 (11)	0.0027 (12)
C10	0.0310 (16)	0.0316 (16)	0.0202 (15)	0.0016 (13)	−0.0051 (12)	0.0009 (12)
C11	0.0297 (15)	0.0308 (15)	0.0223 (14)	−0.0063 (13)	−0.0085 (12)	0.0009 (12)
C12	0.0259 (15)	0.0407 (17)	0.0276 (16)	−0.0063 (13)	0.0002 (12)	−0.0002 (14)
C13	0.0297 (15)	0.0329 (15)	0.0314 (16)	−0.0007 (14)	0.0003 (12)	−0.0032 (14)
C14	0.0409 (18)	0.0352 (17)	0.0364 (18)	0.0033 (15)	0.0027 (14)	0.0041 (14)

### Geometric parameters (Å, °)

**Table:**

Na1—O9	2.382 (3)	O14—H30	0.8500
Na1—O8	2.390 (3)	O14—H29	0.8500
Na1—O1	2.399 (3)	N1—C4	1.370 (4)
Na1—O7	2.453 (3)	N1—H1A	0.8600
Na1—O10	2.466 (3)	N1—H1B	0.8600
Na1—O11	2.670 (3)	N2—C11	1.393 (4)
Na1—Na2	3.3634 (18)	N2—H2A	0.8600
Na2—O10	2.341 (3)	N2—H2B	0.8600
Na2—O13	2.348 (3)	C1—C2	1.377 (4)
Na2—O14	2.380 (3)	C1—C6	1.390 (4)
Na2—O9	2.441 (3)	C2—C3	1.377 (4)
Na2—O12	2.490 (3)	C2—H2	0.9300
Na2—O11	2.516 (3)	C3—C4	1.413 (4)
S1—O1	1.438 (3)	C3—C7	1.502 (5)
S1—O3	1.446 (2)	C4—C5	1.384 (5)
S1—O2	1.464 (2)	C5—C6	1.371 (5)
S1—C1	1.756 (3)	C5—H5	0.9300
S2—O5	1.444 (2)	C6—H6	0.9300
S2—O6	1.457 (2)	C7—H7A	0.9600
S2—O4	1.464 (2)	C7—H7B	0.9600
S2—C8	1.753 (3)	C7—H7C	0.9600
O7—H15	0.8500	C8—C9	1.377 (4)
O7—H16	0.8500	C8—C13	1.394 (4)
O8—H17	0.8499	C9—C10	1.386 (4)
O8—H18	0.8499	C9—H9	0.9300
O9—H19	0.8500	C10—C11	1.414 (4)
O9—H20	0.8500	C10—C14	1.496 (5)
O10—H21	0.8500	C11—C12	1.380 (5)
O10—H22	0.8499	C12—C13	1.382 (5)
O11—H23	0.8500	C12—H12	0.9300
O11—H24	0.8500	C13—H13	0.9300
O12—H25	0.8499	C14—H14A	0.9600
O12—H26	0.8499	C14—H14B	0.9600
O13—H27	0.8500	C14—H14C	0.9600
O13—H28	0.8500		
			
O9—Na1—O8	157.37 (11)	Na1—O11—H24	137.9
O9—Na1—O1	97.10 (10)	H23—O11—H24	106.5
O8—Na1—O1	105.53 (10)	Na2—O12—H25	113.0
O9—Na1—O7	91.27 (9)	Na2—O12—H26	114.1
O8—Na1—O7	91.68 (10)	H25—O12—H26	107.6
O1—Na1—O7	83.80 (9)	Na2—O13—H27	127.7
O9—Na1—O10	85.95 (9)	Na2—O13—H28	96.2
O8—Na1—O10	98.02 (10)	H27—O13—H28	103.9
O1—Na1—O10	78.13 (9)	Na2—O14—H30	120.3
O7—Na1—O10	161.21 (10)	Na2—O14—H29	116.6
O9—Na1—O11	72.53 (9)	H30—O14—H29	104.9
O8—Na1—O11	87.93 (10)	C4—N1—H1A	120.0
O1—Na1—O11	146.41 (10)	C4—N1—H1B	120.0
O7—Na1—O11	127.23 (10)	H1A—N1—H1B	120.0
O10—Na1—O11	69.47 (9)	C11—N2—H2A	120.0
O10—Na2—O13	89.72 (10)	C11—N2—H2B	120.0
O10—Na2—O14	168.83 (10)	H2A—N2—H2B	120.0
O13—Na2—O14	85.43 (10)	C2—C1—C6	120.5 (3)
O10—Na2—O9	87.45 (9)	C2—C1—S1	120.5 (2)
O13—Na2—O9	174.00 (13)	C6—C1—S1	119.0 (2)
O14—Na2—O9	96.40 (9)	C3—C2—C1	121.3 (3)
O10—Na2—O12	99.78 (9)	C3—C2—H2	119.3
O13—Na2—O12	99.86 (12)	C1—C2—H2	119.3
O14—Na2—O12	90.97 (9)	C2—C3—C4	118.5 (3)
O9—Na2—O12	85.84 (9)	C2—C3—C7	121.5 (3)
O10—Na2—O11	74.15 (10)	C4—C3—C7	120.0 (3)
O13—Na2—O11	99.73 (13)	N1—C4—C5	121.4 (3)
O14—Na2—O11	96.74 (10)	N1—C4—C3	119.5 (3)
O9—Na2—O11	74.40 (9)	C5—C4—C3	119.1 (3)
O12—Na2—O11	159.44 (11)	C6—C5—C4	122.0 (3)
O1—S1—O3	113.08 (18)	C6—C5—H5	119.0
O1—S1—O2	111.53 (15)	C4—C5—H5	119.0
O3—S1—O2	110.55 (16)	C5—C6—C1	118.5 (3)
O1—S1—C1	107.05 (15)	C5—C6—H6	120.7
O3—S1—C1	106.96 (15)	C1—C6—H6	120.7
O2—S1—C1	107.35 (14)	C3—C7—H7A	109.5
O5—S2—O6	112.54 (15)	C3—C7—H7B	109.5
O5—S2—O4	112.49 (15)	H7A—C7—H7B	109.5
O6—S2—O4	110.33 (15)	C3—C7—H7C	109.5
O5—S2—C8	106.57 (15)	H7A—C7—H7C	109.5
O6—S2—C8	107.40 (15)	H7B—C7—H7C	109.5
O4—S2—C8	107.16 (14)	C9—C8—C13	120.7 (3)
S1—O1—Na1	139.61 (15)	C9—C8—S2	120.4 (2)
Na1—O7—H15	111.2	C13—C8—S2	119.0 (2)
Na1—O7—H16	109.9	C8—C9—C10	121.1 (3)
H15—O7—H16	105.1	C8—C9—H9	119.4
Na1—O8—H17	93.5	C10—C9—H9	119.4
Na1—O8—H18	110.3	C9—C10—C11	118.3 (3)
H17—O8—H18	108.1	C9—C10—C14	121.3 (3)
Na1—O9—Na2	88.42 (8)	C11—C10—C14	120.4 (3)
Na1—O9—H19	103.2	C12—C11—N2	120.9 (3)
Na2—O9—H19	122.6	C12—C11—C10	119.7 (3)
Na1—O9—H20	120.5	N2—C11—C10	119.4 (3)
Na2—O9—H20	115.9	C11—C12—C13	121.6 (3)
H19—O9—H20	105.6	C11—C12—H12	119.2
Na2—O10—Na1	88.77 (9)	C13—C12—H12	119.2
Na2—O10—H21	127.5	C12—C13—C8	118.5 (3)
Na1—O10—H21	100.8	C12—C13—H13	120.7
Na2—O10—H22	105.1	C8—C13—H13	120.7
Na1—O10—H22	133.3	C10—C14—H14A	109.5
H21—O10—H22	104.7	C10—C14—H14B	109.5
Na2—O11—Na1	80.80 (8)	H14A—C14—H14B	109.5
Na2—O11—H23	119.3	C10—C14—H14C	109.5
Na1—O11—H23	101.9	H14A—C14—H14C	109.5
Na2—O11—H24	110.2	H14B—C14—H14C	109.5
			
O9—Na1—Na2—O10	147.11 (13)	O12—Na2—O10—Na1	107.97 (9)
O8—Na1—Na2—O10	−60.55 (13)	O11—Na2—O10—Na1	−51.87 (9)
O1—Na1—Na2—O10	58.64 (11)	O9—Na1—O10—Na2	−23.26 (9)
O7—Na1—Na2—O10	152.54 (15)	O8—Na1—O10—Na2	134.33 (10)
O11—Na1—Na2—O10	−105.07 (12)	O1—Na1—O10—Na2	−121.39 (10)
O9—Na1—Na2—O13	−175.12 (17)	O7—Na1—O10—Na2	−105.3 (3)
O8—Na1—Na2—O13	−22.78 (18)	O11—Na1—O10—Na2	49.60 (9)
O1—Na1—Na2—O13	96.41 (16)	O10—Na2—O11—Na1	47.38 (8)
O7—Na1—Na2—O13	−169.69 (17)	O13—Na2—O11—Na1	134.30 (10)
O10—Na1—Na2—O13	37.77 (16)	O14—Na2—O11—Na1	−139.21 (9)
O11—Na1—Na2—O13	−67.30 (17)	O9—Na2—O11—Na1	−44.41 (8)
O9—Na1—Na2—O14	−47.85 (12)	O12—Na2—O11—Na1	−27.9 (3)
O8—Na1—Na2—O14	104.49 (14)	O9—Na1—O11—Na2	46.39 (8)
O1—Na1—Na2—O14	−136.33 (12)	O8—Na1—O11—Na2	−145.20 (10)
O7—Na1—Na2—O14	−42.43 (17)	O1—Na1—O11—Na2	−29.7 (2)
O10—Na1—Na2—O14	165.03 (14)	O7—Na1—O11—Na2	124.26 (11)
O11—Na1—Na2—O14	59.97 (13)	O10—Na1—O11—Na2	−45.84 (8)
O8—Na1—Na2—O9	152.34 (13)	O1—S1—C1—C2	116.4 (3)
O1—Na1—Na2—O9	−88.48 (11)	O3—S1—C1—C2	−5.1 (3)
O7—Na1—Na2—O9	5.42 (14)	O2—S1—C1—C2	−123.7 (3)
O10—Na1—Na2—O9	−147.11 (13)	O1—S1—C1—C6	−62.7 (3)
O11—Na1—Na2—O9	107.82 (12)	O3—S1—C1—C6	175.8 (3)
O9—Na1—Na2—O12	62.13 (11)	O2—S1—C1—C6	57.2 (3)
O8—Na1—Na2—O12	−145.53 (11)	C6—C1—C2—C3	0.9 (5)
O1—Na1—Na2—O12	−26.35 (10)	S1—C1—C2—C3	−178.2 (2)
O7—Na1—Na2—O12	67.56 (14)	C1—C2—C3—C4	−1.1 (5)
O10—Na1—Na2—O12	−84.98 (11)	C1—C2—C3—C7	179.2 (3)
O11—Na1—Na2—O12	169.95 (11)	C2—C3—C4—N1	−180.0 (3)
O9—Na1—Na2—O11	−107.82 (12)	C7—C3—C4—N1	−0.2 (5)
O8—Na1—Na2—O11	44.52 (13)	C2—C3—C4—C5	0.0 (5)
O1—Na1—Na2—O11	163.70 (12)	C7—C3—C4—C5	179.7 (3)
O7—Na1—Na2—O11	−102.39 (15)	N1—C4—C5—C6	−178.6 (3)
O10—Na1—Na2—O11	105.07 (12)	C3—C4—C5—C6	1.4 (5)
O3—S1—O1—Na1	−75.9 (3)	C4—C5—C6—C1	−1.7 (5)
O2—S1—O1—Na1	49.4 (3)	C2—C1—C6—C5	0.5 (5)
C1—S1—O1—Na1	166.6 (2)	S1—C1—C6—C5	179.6 (2)
O9—Na1—O1—S1	−115.0 (3)	O5—S2—C8—C9	−22.7 (3)
O8—Na1—O1—S1	64.5 (3)	O6—S2—C8—C9	−143.5 (3)
O7—Na1—O1—S1	154.5 (3)	O4—S2—C8—C9	98.0 (3)
O10—Na1—O1—S1	−30.6 (3)	O5—S2—C8—C13	157.2 (2)
O11—Na1—O1—S1	−46.0 (4)	O6—S2—C8—C13	36.4 (3)
Na2—Na1—O1—S1	−68.0 (3)	O4—S2—C8—C13	−82.1 (3)
O8—Na1—O9—Na2	−78.9 (3)	C13—C8—C9—C10	−0.9 (5)
O1—Na1—O9—Na2	99.74 (9)	S2—C8—C9—C10	179.0 (2)
O7—Na1—O9—Na2	−176.35 (9)	C8—C9—C10—C11	−1.5 (5)
O10—Na1—O9—Na2	22.25 (8)	C8—C9—C10—C14	178.3 (3)
O11—Na1—O9—Na2	−47.48 (9)	C9—C10—C11—C12	2.6 (4)
O10—Na2—O9—Na1	−23.48 (9)	C14—C10—C11—C12	−177.2 (3)
O13—Na2—O9—Na1	38.5 (12)	C9—C10—C11—N2	−177.7 (3)
O14—Na2—O9—Na1	146.00 (9)	C14—C10—C11—N2	2.5 (4)
O12—Na2—O9—Na1	−123.48 (9)	N2—C11—C12—C13	179.0 (3)
O11—Na2—O9—Na1	50.77 (9)	C10—C11—C12—C13	−1.3 (5)
O13—Na2—O10—Na1	−152.07 (12)	C11—C12—C13—C8	−1.1 (5)
O14—Na2—O10—Na1	−87.9 (5)	C9—C8—C13—C12	2.2 (4)
O9—Na2—O10—Na1	22.63 (8)	S2—C8—C13—C12	−177.7 (2)

### Hydrogen-bond geometry (Å, °)

**Table:**

*D*—H···*A*	*D*—H	H···*A*	*D*···*A*	*D*—H···*A*
N1—H1A···O4^i^	0.86	2.30	3.046 (4)	145
N2—H2A···O1^ii^	0.86	2.30	3.066 (4)	149
O7—H15···N2^iii^	0.85	2.13	2.929 (4)	157
O7—H16···O2^iv^	0.85	1.98	2.809 (3)	166
O8—H17···O12^v^	0.85	2.29	2.800 (4)	119
O8—H18···O6	0.85	2.14	2.989 (4)	171
O9—H19···O3^vi^	0.85	2.00	2.814 (4)	160
O9—H20···O8^vi^	0.85	2.06	2.911 (4)	175
O10—H21···O2	0.85	1.99	2.831 (3)	172
O10—H22···O14^vii^	0.85	2.01	2.812 (4)	157
O11—H23···O4	0.85	2.05	2.895 (4)	175
O11—H24···O5^vi^	0.85	2.03	2.872 (4)	169
O12—H25···O7^viii^	0.85	1.95	2.794 (3)	175
O12—H26···O2^vi^	0.85	2.09	2.889 (4)	157
O13—H27···O6^viii^	0.85	2.12	2.969 (4)	175
O13—H28···O5^ix^	0.85	2.32	2.883 (4)	124
O14—H29···O6^ix^	0.85	2.04	2.837 (3)	156
O14—H30···O4^vi^	0.85	1.95	2.790 (3)	171

Symmetry codes: (i) −*x*, *y*+1/2, −*z*; (ii) −*x*, *y*−1/2, −*z*+1; (iii) −*x*, *y*+1/2, −*z*+1; (iv) *x*, *y*, *z*+1; (v) *x*+1, *y*, *z*+1; (vi) *x*−1, *y*, *z*; (vii) *x*+1, *y*, *z*; (viii) *x*, *y*, *z*−1; (ix) *x*−1, *y*, *z*−1.
